# The neurocognitive consequences of the wandering mind: a mechanistic account of sensory-motor decoupling

**DOI:** 10.3389/fpsyg.2013.00725

**Published:** 2013-10-14

**Authors:** Julia W. Y. Kam, Todd C. Handy

**Affiliations:** Attentional Neuroscience Lab, Department of Psychology, University of British ColumbiaVancouver, BC, Canada

**Keywords:** mind wandering, decoupling, sensory attenuation, cognitive attenuation, executive resources, neurocognitive pathologies

## Abstract

A unique human characteristic is our ability to mind wander – a state in which we are free to engage in thoughts that are not directly tied to sensations and perceptions from our immediate physical environment. From a neurocognitive perspective, it has been proposed that during mind wandering, our executive resources are decoupled from the external environment and directed to these internal thoughts. In this review, we examine an underappreciated aspect of this phenomenon – attenuation of sensory-motor processing – from two perspectives. First, we describe the range of widespread sensory, cognitive and motor processes attenuated during mind wandering states, and how this impacts our neurocognitive processing of external events. We then consider sensory-motor attenuation in a class of clinical neurocognitive disorders that have ties to pathological patterns of decoupling, reviews suggesting that mind wandering and these clinical states may share a common mechanism of sensory-motor attenuation. Taken together, these observations suggest the sensory-motor consequences of decoupled thinking are integral to normal and pathological neurocognitive states.

Mind wandering is the ubiquitous phenomenon in which our minds drift away from perceptual and cognitive demands of the immediate external environment to focus on the internal milieu. From planning for the weekend to fantasizing about our next vacation, we easily get lost in our own thoughts, especially when performing well-practiced tasks such as driving or washing dishes. In these instances, our minds become decoupled from stimulus events in the external environment, a regular and periodic experience that occupies a notable portion of our mental life (e.g., Klinger and Cox, 1987; Smallwood and Schooler, 2006; Killingsworth and Gilbert, 2010). Indeed, our proclivity to mind wander is sufficiently hard-wired that despite the best of our will power, these “decoupled” thoughts occur whether we want them to or not (Braboszcz et al., 2010).

A key issue in the scientific study of mind wandering concerns understanding the qualitative content of decoupled thoughts (McVay et al., 2009; Killingsworth and Gilbert, 2010; Smallwood et al., 2011). For example, in terms of what our minds focus on when they wander, we are more likely to think about the future than the past or present, an effect that has been linked to strategic planning (Smallwood et al., 2011). These task-unrelated thoughts are also more likely to concern personal issues instead of unfocused daydreams (McVay et al., 2009), underscoring their utility. Further, when mind wandering in their daily lives, individuals also report being less happy, regardless of whether their thoughts were unpleasant or neutral (Killingsworth and Gilbert, 2010). These findings and others have all helped elucidate the nature of thoughts inside the wandering mind.

Notably, however, the consequences of mind wandering extend beyond the qualitative content of decoupled thinking itself, and the adaptive value it provides our species (for a review, see Schooler et al., 2011). In particular, there has been growing interest in understanding the impact decoupled thinking has on how we process and respond to stimuli in the external environment (e.g., Smallwood et al., 2008; O’Connell et al., 2009; Barron et al., 2011; Stawarczyk et al., 2011), an issue that is pressing for two central reasons. From a basic cognitive neuroscience perspective, mind wandering is now being recognized as a novel form of attentional selection, where our attention to the outside world is disrupted, such that we no longer highlight or “select” certain external stimuli for preferential neurocognitive processing (e.g., Kam et al., 2011). Likewise, from a clinical perspective, abnormal patterns of mind wandering and their effects on attention to the external environment have been implicated in conditions such as attention deficit/hyperactivity disorder (ADHD) and depression (e.g., Sonuga-Barke and Castellanos, 2007). Taken together, these considerations have placed a premium on elucidating the details of how mind wandering alters our neurocognitive processing of external stimulus events.

Importantly, discussion of these sensory-motor effects also helps to illuminate current models of mind wandering themselves. To the point, there has been a growing debate over exactly how sensory-motor processing should be affected as one slips into a mind wandering state. On the one hand, the “decoupling hypothesis” predicts that the maintenance of self-generated thoughts should attenuate the sensory-motor processing of external events, owing to the fact that these thoughts, and the subjective experiences they invoke in particular, rely on the same domain-specific processes engaged by events in the external world (e.g., Smallwood and Schooler, 2006; Schooler et al., 2011; Smallwood, 2013). Consistent with this view, visual (e.g., Kosslyn et al., 1997), auditory (e.g., Zatorre and Halpern, 2005), and motor imagery (e.g., Decety, 1996) have all been shown to activate the majority of domain-specific regions of cortex activated during actual sensory-motor engagement with the outside world. In this view, self-generated thoughts would have the same impact of sensory-motor regions of cortex as active imagery itself, leading to a general attenuation of processing of external events during mind wandering episodes.

On the other hand, the “executive failure hypothesis” proposes that mind wandering is the direct result of an inability to maintain selective attention to current task-relevant goals in the external environment (e.g., McVay and Kane, 2009, 2010; Smallwood, 2013). This has led to the suggestion that, when these executive processes fail during mind wandering, the concomitant “release” of selective attentional control over sensory-motor processing should lead to either no changes, or perhaps even *increases,* in task-irrelevant external stimulus processing (Smallwood, 2013). Consistent with this perspective has been the finding that when executive processes such as working memory are overloaded by extreme task demands, selective attention does appear to fail, producing increases in the processing of task-irrelevant information in the external environment (e.g., de Fockert et al., 2001). With mind wandering, the idea here is that when executive processes fail and a mind wandering episode is initiated, task-irrelevant external events that were heretofore suppressed via top-down executive control are now processed to a greater extent.

Given these competing predictions regarding how mind wandering should impact sensory-motor processing, our review provides direct support for the sensory-motor predictions of the “decoupling hypothesis.” While it remains uncertain whether the attenuation effects we report are in fact driven by engagement of these sensory-motor processes by self-generated thought as the hypothesis predicts, the attenuation itself is consistent with that prediction. With respect to the “executive failure hypothesis,” however, we believe the prediction of unchanged or increased sensory-motor processing during mind wandering (Smallwood, 2013) may not in fact be tenable. Instead, one can reconcile the “executive failure hypothesis” with widespread sensory motor attenuation if one assumes that once engaged in self-generated thought, this would have an attenuating impact on sensory-motor processing regardless of whether selective task-related attention is released. In both cases, however, consideration of the sensory-motor consequences of mind wandering help illuminate important questions to consider for models of mind wandering in general.

Overall, the sensory-motor attenuation effects of mind wandering ripple widely, extending to a broad array of neurocognitive functions and systems. Below, we begin by reviewing these effects, concerning the idea that decoupled thoughts are associated with an attenuation of processing in neural systems that are often engaged or “coupled” with the external sensory-motor environment in order to adaptively guide our behavior. We then consider pathologies of neurocognitive function that may be tied to abnormal patterns of decoupling. Following this, we conclude by pointing out methodological and theoretical considerations and directions for future investigations.

## WHICH NEUROCOGNITIVE FUNCTIONS ARE ATTENUATED DURING THE DECOUPLED STATE?

Attenuation of sensory-motor processing of stimuli in the environment appears to play an important role in facilitating the production and maintenance of ongoing decoupled thoughts (e.g., Schooler et al., 2011; Smallwood, 2013). Given the myriad of information that can capture our attention and the limited resources available, normal functioning would require that we suppress goal-irrelevant events in the immediate environment to focus on the relevant ones. As support for the executive function model (Smallwood and Schooler, 2006), we summarize evidence suggesting that during mind wandering, executive resources are decoupled from our immediate environment and directed them to inner streams of thoughts via this global sensory-motor attenuation. Based on our review of the literature, we suggest that the attenuation of external processing plays an important role in mind wandering, an idea that is supported by a recent theoretical paper (Smallwood, 2013).

Before detailing specific effects, however, there are at least two key points to consider. First, the attenuating effects of mind wandering on sensory-motor processing are multi-faceted, in that they occur at multiple levels of processing and disrupt various types of stimulus-related responses. Gathering evidence from both neural and behavioral investigations, mind wandering has been associated with reduction in sensory and cognitive processing of external inputs, as well as changes in the accuracy and variability of response in attention and motor task performance. Second, for any given system or process attenuated by mind wandering, the magnitude of the effect can be graded. That is, the effects can range from partial attenuation, reducing the extent of stimulus processing but not eliminating it, to full-blown attenuation of external stimulus processing. In this section, we outline what has been discovered about the functional consequences of mind wandering in terms of sensory-motor attenuation, with a particular emphasis on the event-related potential (ERP) studies we conducted over the last several years.

### MIND WANDERING ATTENUATES COGNITIVE PROCESSING

Attentional lapses have been shown to disrupt behavioral performance, yet the neural mechanism underlying this process has been unclear. To examine this issue, Weissman et al. (2006) had participants perform a global/local task during which their reaction times (RTs) and neural activity (i.e., functional magnetic resonance imaging, fMRI) were recorded. Using a behavioral measure, they considered slower RTs to relevant stimuli as an index of attentional lapses. During these momentary lapses in attention, cortical regions involved in attentional orienting, working memory and conflict resolution were found to down regulate (Weissman et al., 2006).

Given these findings, to what extent do the reduced levels of activation in the brain correspond to a dampened cognitive analysis of external events during mind wandering episodes? Based on past observations that simple tasks elicit high levels of mind wandering (Teasdale et al., 1993; Giambra, 1995), our study asked participants to perform the sustained attention to response task (SART; Robertson et al., 1997), where they simply responded to frequent non-targets and withheld their response to an infrequent target (Smallwood et al., 2008). To examine how SART performance was modulated by attention to task, mind wandering episodes were defined by two approaches – an objective behavioral error measure, indexing the inability to withhold responses to the infrequent target, and a subjective measure in which subjects verbally report their task-related attention state in the moment as “on-task” or “mind wandering.” We recorded ERPs during the task, and focused our analysis on the P3 ERP component elicited by the frequent non-targets, a component which indexes the depth of cognitive analysis of stimulus events (Polich, 2007). Specifically, we examined the P3 as a function of whether the frequent non-target preceded “on-task” or “mind wandering” reports.

**FIGURE 1:**
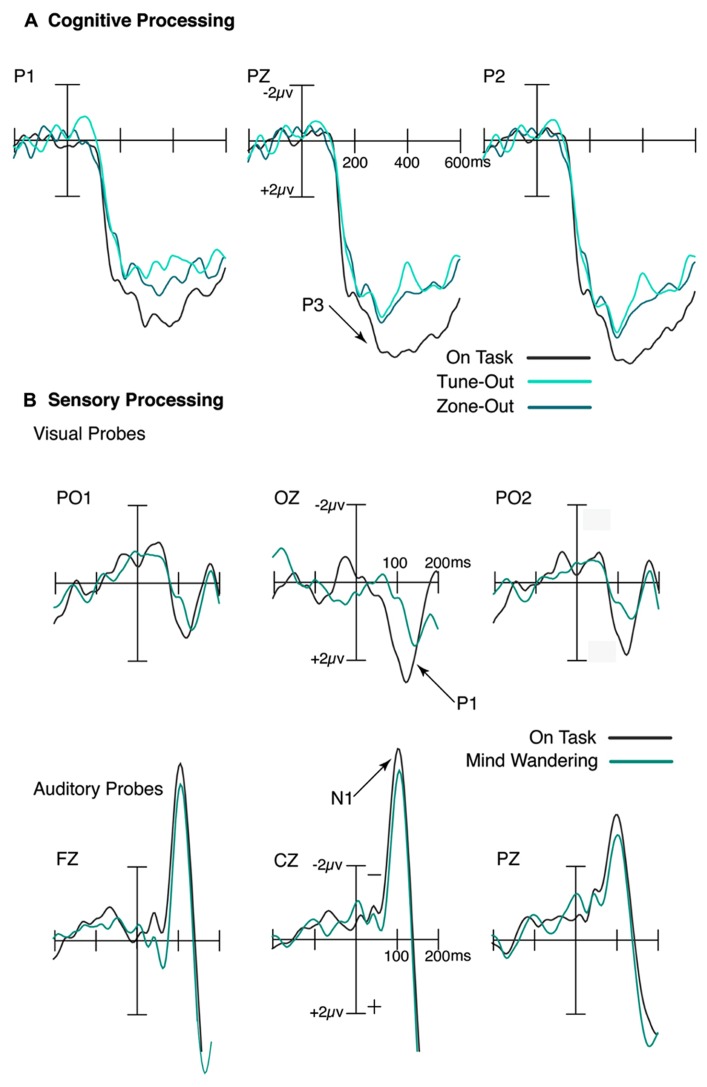
**Attenuation of sensory and cognitive processing during mind wandering.** **(A)** During decoupled thoughts, the cognitive response to targets, as indexed by the P3 ERP component, was significantly reduced. **(B)** Similarly, the sensory processing of both auditory and visual stimuli, as indexed by the N1 and P1 respectively, was attenuated in periods immediately preceding mind wandering relative to on-task reports.

We found an attenuation of the P3 to the frequent stimuli in the interval immediately preceding subjective reports of mind wandering episodes, as shown in **Figure 1A**. Further, a comparable P3 reduction was observed in the period immediately prior to performance errors. These findings indicate that the extent of cognitive processing during episodes of mind wandering appears to imitate periods leading up to performance errors, suggesting the overall lower level of cognitive analysis of external events may be tightly linked to disruptions in task performance (Smallwood et al., 2008). As such, it appears that cognitive processing was indeed attenuated during episodes of mind wandering as indexed by both objective and subjective measures, and that this attenuation effect can be accurately indexed by subjective reports of task-related attention (Smallwood et al., 2008).

These findings were replicated by O’Connell et al. (2009), who attempted to identify neural signatures of lapses in sustained attention. In their study, participants were asked to identify the “target” visual stimulus, which was presented for a longer duration relative to the standard visual stimulus. They examined the ERP responses to the targets prior to behavioral measures of attentional lapses, which were indexed by the failure to detect targets. They found a reduced P3 and contingent-negative variation (CNV), an ERP component that reflects the magnitude of an anticipatory response to an expected stimulus, for up to 4 s prior to missed targets. This implies a disruption in both target anticipation and disengagement from ongoing task while in the decoupled state.

If episodes of mind wandering attenuate cognitive responses, however, is this attenuation effect restricted to task-relevant processing only, or is there a general attentuation on cognitive processing of external inputs regardless of their relevance? To address that question, Barron et al. (2011) asked participants to perform a visual oddball task, which required them to manually respond to the task-relevant oddball, and ignore the task-irrelevant novel stimuli that acted as distractors, both of which occurred at low frequency. The participants were categorized into three groups (high, medium, low) depending on the frequency of their task-unrelated thoughts, as retrospectively reported by them on a questionnaire upon task completion. Relative to frequent standard stimuli, they found a reduced P3 to both the task-irrelevant novel distractor, thought to reflect attentional processing of rare stimuli, as well as the task-relevant oddball stimuli, thought to reflect cognitive processing in general and maintenance of task-relevant stimuli in working memory. Therefore, mind wandering appears to disrupt cognitive level processing of infrequent stimuli regardless of their relevance to the ongoing task.

Yet, it remains unclear whether the attenuating effects of mind wandering captured by the P3 are reflective of the relatively impoverished nature of the stimuli used in these previous studies, or whether we would observe a similar disruptive effect with affectively salient stimuli while being decoupled from the external environment. In a recent study, we examined whether the cognitive analysis of naturalistic stimuli with some measure of affective saliency changes as a function of our attention to task (Kam et al., 2013a). Specifically, we recorded ERPs while subjects viewed photos of hands in painful or neutral situations, and reported their attention state as “on-task” or “mind wandering” when probed at random intervals. We then compared the ERPs to painful and neutral images in the interval immediately preceding “on-task” vs. “mind wandering” reports.

In the first experiment, we observed a reduced P3 to the painful stimuli during the interval prior to mind wandering reports relative to on-task reports. Likewise, our second experiment showed a corresponding behavioral effect, that is, a reduction of painfulness ratings of painful stimuli during mind wandering episodes (Kam et al., 2013b). Together, our findings suggest that mind wandering reduces our cognitive evaluation of external inputs even for affectively salient stimuli. The variety of stimuli used across these studies to examine the attenuating effects of mind wandering indicate that the association between mind wandering and disrupted cognitive analysis of external events appears to occur across different contextual representations.

### MIND WANDERING ATTENUATES SENSORY PROCESSING

These above findings converge on the notion that mind wandering attenuates cognitive responses to visual stimuli. Nevertheless, are these effects also observed earlier in the processing stream, during the initial, sensory-evoked responses to external stimulus inputs? To examine this question, we recorded ERPs while participants performed a variation of the SART, which included two task-irrelevant stimuli – a visual probe presented in the periphery and an auditory tone. Participants were also asked to verbally report their attention state throughout the task. We then examined the N1 (indicative of sensory processing in the auditory domain) in response to the auditory tone, and the P1 (indicative of sensory visual processing) in response to the visual probe as a function of whether they preceded “on-task” or “mind wandering” reports.

Our results indicated a reduction in sensory-evoked cortical activity to task irrelevant probes in the interval prior to mind wandering reports (Kam et al., 2011). Specifically, the N1 and P1, in response to the auditory and visual probes respectively, were both reduced during episodes of mind wandering relative to on-task, as shown in **Figure 1B**. Moreover, that the ongoing task was in the visual modality suggests that the disruptive effects of mind wandering were not modality-specific. This finding is consistent with two fMRI studies (Weissman et al., 2006, 2009) that found reduced sensory responses in cortex to task-relevant stimuli during brief lapses of attention.

Given a reduction in auditory sensory processing during mind wandering states, are we also less able to detect a change in the external stimulus in the auditory domain? In one study, participants were asked to focus on their breath while they passively listened to frequent and rare auditory tones (as in a passive oddball task). They were also asked to report when they noticed they were mind wandering (Braboszcz and Delorme, 2011). The detection of rare auditory tones as indexed by the mismatch negativity (MMN) component was attenuated during episodes of mind wandering compared to episodes of on-task. That the process underlying this MMN component is considered as automatic and pre-attentive sensory level perception suggests that mind wandering is not only associated with reduced sensory processing, but also a reduced sensory detection of change in auditory perception (Braboszcz and Delorme, 2011).

Collectively, these studies indicate that mind wandering attenuates the same visual sensory response in cortex that are affected by top-down selective attention (e.g., Mangun and Hillyard, 1991; Heinze et al., 1994; Woldorff et al., 1997). As argued by Kam et al. (2011), this supports the notion that attentional control of sensory processing in visual cortex is modulated by two control systems operating in parallel (Dosenbach et al., 2008), one associated with rapid shifts of selective visual attention (e.g., Corbetta et al., 1991; Hopfinger et al., 2000), and a second one associated with slower fluctuations in task-related attention (e.g., Sonuga-Barke and Castellanos, 2007). The attenuation of sensory responses associated with this latter form of attention is what appears to facilitate our internal streams of thoughts during periods of decoupled thinking, or mind wandering.

### MIND WANDERING DISRUPTS ATTENTION AND MOTOR TASK PERFORMANCE

**FIGURE 2:**
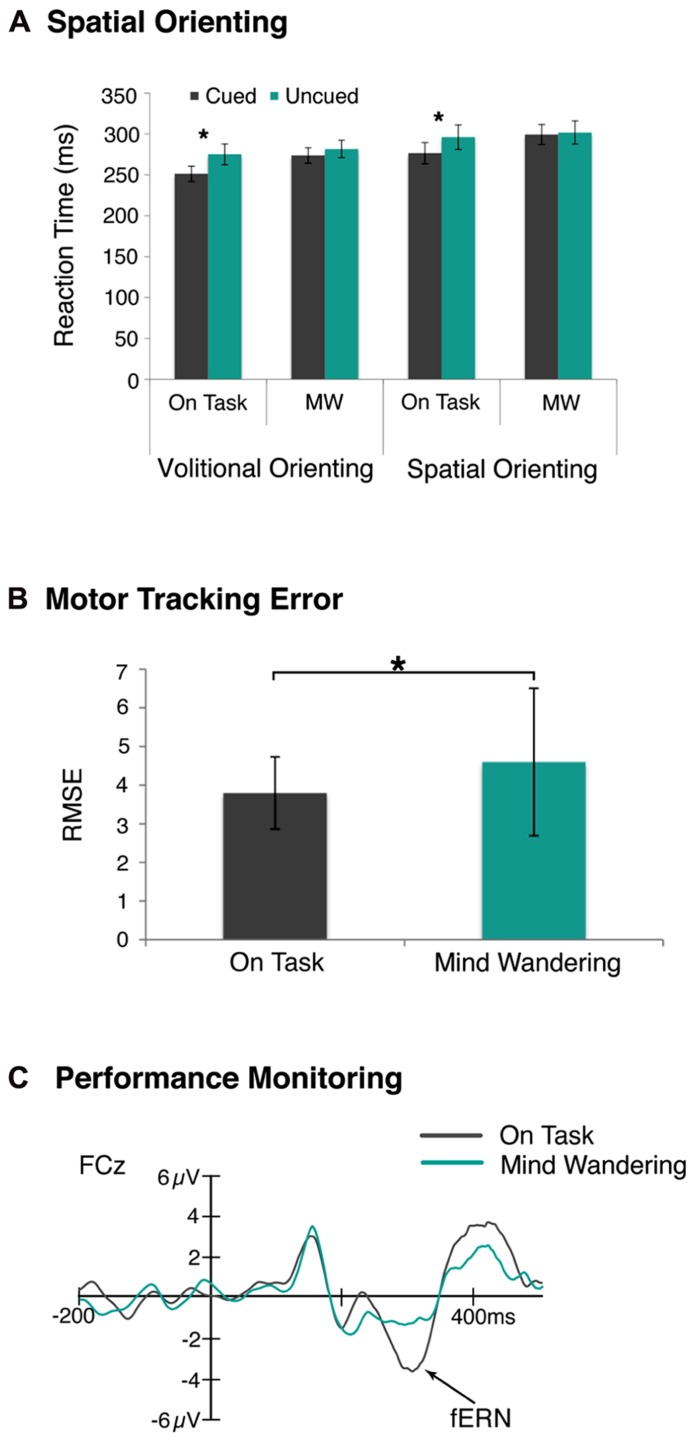
**Disruption of attention and motor task performance during mind wandering.** **(A)** RTs were significantly faster to cued trials compared to uncued trials during on-task state only, suggesting attentional orienting was disrupted during mind wandering state. This was observed in both volitional and reflexive orienting tasks. **(B)** The tracking error, as measured by the root mean square error, in a visuomotor tracking task was significantly greater during decoupled thoughts. **(C)** Likewise, performance monitoring was disrupted, as indexed by a reduced fERN component, during episodes of mind wandering compared to on-task *p < 0.05.

Given that our sensory and cognitive processing of environmental stimuli attenuates during mind wandering, it is not surprising that much behavioral evidence confirms the intuition that mind wandering disrupts our attentional systems and motor task performance as well. With respect to the former, the selective attention model assumes that the spotlight is always on (e.g., Posner, 1980), but are there times when we turn off that attentional spotlight, for example when we are mind wandering? To address this question, we examined whether volitional attentional functions change as we drift in and out of mind wandering states, and if so, how this compares to the impact of mind wandering on more reflexive attentional functions (Kam et al., 2013a). Participants performed one of two spatial orienting tasks, and we compared RTs to cued and uncued trials as a function of the reported attention state. As shown in **Figure 2A**, both forms of visual-spatial attentional orienting were disrupted during episodes of mind wandering compared to on-task. This finding indicates that we do indeed turn off the attentional spotlight when we mind wander.

Based on the widespread decoupling of executive functions from the sensory-motor environment when mind wandering, is there also a similar disengagement of processes associated with behavioral responses? Much evidence now converges on the notion that mind wandering impacts behavioral performance. For instance, increased RT and errors have been reported during periods of mind wandering (Robertson et al., 1997; Smallwood et al., 2004, 2006; Cheyne et al., 2006; McVay and Kane, 2009). In addition to the global change in RT associated with this attentional state, Stawarczyk et al. (2011) also found an increase in intra-individual RT variability. Consistent with the predictions made by Sonuga-Barke and Castellanos (2007), these findings suggest that individuals not only respond more impulsively (as indexed by the quicker RT) but also with less stability (as indexed by the increased variability in RT) when we are disengaged from the external environment.

Yet it is unclear whether this form of disengagement is a direct consequence of disrupted attention and sensory/cognitive processing, or whether the behavioral disengagement occurs even when sensory and other cognitive level processing are intact. In order to address this, we first determined whether motor behavior was actually disrupted during mind wandering by asking participants to track a moving target across the screen and occasionally report their attentional state (Kam et al., 2012). The magnitude of tracking error was then examined as a function of whether they preceded on-task or mind wandering reports. We observed that tracking error was in fact greater in the visuomotor task during mind wandering relative to on-task state, as measured by root mean squared error, as shown in **Figure 2B**.

To ascertain whether this effect occurred independent of disruptions earlier or later in the processing stream, we then examined performance monitoring as indexed by the feedback error related negativity (fERN) component (Miltner et al., 1997; Holroyd and Krigolson, 2007) as a function of the reported attention state. Participants were asked to perform a time-estimation task with trial-by-trial feedback, while we recorded their fERN to the feedback, and occasionally asked them to report their attention state. We found disrupted performance monitoring, as indexed by a reduced fERN, during episodes of mind wandering, as shown in **Figure 2C**. Importantly, this effect could not be ascribed to sensory or cognitive responses, as these responses interacted differently with mind wandering episodes suggesting a functional dissociation between these responses and the fERN. These results together suggest that mind wandering disengages us from monitoring and adjusting our behavioral outputs independent of earlier and later processing (Kam et al., 2012). Further, the disrupted monitoring of our task performance appears to at least partially account for the robust relationship observed between mind wandering and performance failure in simple attention and motor tasks.

## ARE ANY NEUROCOGNITIVE FUNCTIONS PRESERVED DURING THE DECOUPLED STATE?

Thus far, the studies we discussed suggest that both sensory and cognitive responses are attenuated during periods of mind wandering, effects that are manifest in attentional and behavioral performance as well. Nevertheless, we seem quite capable of responding to the external environment even when our minds have wandered. This suggests that some aspects of attentional processing of stimulus in the environment may be preserved during mind wandering, functions that allow us to adaptively respond to stimuli in the external environment despite our cognitively decoupled state.

One potential candidate is deviance detection (e.g., Näätänen et al., 1978; Escera et al., 1998) – the extent to which we perceive a rare or unexpected stimulus in the environment. To explore this issue, we presented participants with task-irrelevant auditory stimuli in the background while they read a book, and asked them to occasionally report their attention state. We found that both frequent, standard tones and infrequent, deviant tones elicited an N1 in our participants (Kam et al., 2013a). More importantly, while the N1 in response to standard tones was greater when attention is on-task relative to mind wandering, as would be expected based on the aforementioned study (Kam et al., 2011), the magnitude of N1 in response to the deviant tones did not significantly differ between the two attentional states. This suggests that despite their irrelevance to the ongoing task, the sensory processing of rare, deviant events in the environment was in fact preserved during mind wandering. While this appears to stand in contrast with the findings of disrupted deviance detection, as indexed by a reduced MMN (Braboszcz and Delorme, 2011), several reasons may account for this discrepancy. For example, the nature of the ongoing task, the methodology of experience sampling and the amount of data considered to be reflective of the reported attentional state, are only a few differences between these two studies that may have led to the disparate results, all of which are detailed in the study by Kam et al. (2013a).

These sets of findings suggest that the magnitude of sensory-evoked cortical activity during mind wandering episodes may be dependent upon the nature and importance of the external stimulus. That is, if external and internal stimuli compete for executive resources (Smallwood and Schooler, 2006), much in the way stimuli in our visual field compete for selective spatial attention (e.g., Posner, 1980), then perhaps there is a constant evaluation of which stimulus is more worthy of our attention at any given time. In particular, this is consistent with views on competition of available resources in the context of selective attention. For example, given a single, finite pool of attentional resources, one model suggests that we can only attend to one stream of input at a time, and therefore few if any resources remain for the unselected input (Kahneman, 1973). Alternatively, another perspective relates to the integrated competition hypothesis, which suggests that visual objects compete to be represented in various neurocognitive systems (Desimone and Duncan, 1995). Once selected, the perceptual processing of the selected object is prioritized by the cooperation of multiple brain systems working together to analyze the different properties of that object (Duncan et al., 1997). More generally, this competition process was proposed to occur in not only the sensory domain as suggested by Desimone and Duncan (1995), but also in the emotion and memory domains (Miller and Cohen, 2001).

Importantly, these views apply to the competition between internal and external inputs as well (Smallwood and Schooler, 2006). On one hand, it has been suggested that our minds are shielded from mundane sensory events to facilitate internal thoughts (Schooler et al., 2011). However, when an unexpected event occurs in the environment, one that is potentially dangerous, we may ascribe that event with higher priority and consequently shift our attention to the external environment. Taken together, our minds may engage in an ongoing evaluation of the importance of both external and internal stimuli; after a decision is made, our attention is then allocated accordingly. Even when our mind is wandering, it appears we are still clever about how we selectively disengage from the external environment – we remain vigilant for deviant or unusual events that may require re-engaging our neurocognitive resources with the external sensory-motor environment.

## NEUROCOGNITIVE PATHOLOGIES AND SENSORY-MOTOR ATTENUATION

Until now, our discussion of mind wandering has concerned its association with the attenuation of sensory-motor processing of external stimulus inputs. As we have noted above, the effects we review in this regard are not only consistent with the predictions of the “decoupling hypothesis” of mind wandering, but they suggest that revisions may be in order for sensory-motor predictions ascribed to the “executive failure hypothesis” (e.g., Smallwood, 2013). However, the value of reviewing the impacts of mind wandering on sensory-motor processing goes beyond helping clarify and inform on extant models of mind wandering itself.

In particular, a variety of different neurocognitive disorders – such as depression and attention deficit/hyperactivity disorder – have been associated with abnormal or pathological patterns of sensory-motor attenuation. This suggests that sensory-motor attenuation is not necessarily specific to mind wandering itself, but rather, reflects a general capacity or mechanism we have to insulate ourselves from stimulus events in the outside world as necessary or so desired. In the following section, we examine sensory-motor attenuation as it relates to clinical disorders of neurocognitive function, with the goal of highlighting two different ways in which it can be engaged in a non-normative manner. The first concerns the profile of attenuation itself, or the range of neurocognitive processes attenuated and the magnitude of those effects. The second concerns the prevalence of sensory-motor attenuation. Taken together, we suggest that these two sides to abnormal sensory-motor attenuation provide a novel perspective on an otherwise broad class of neurocognitive pathologies.

### ABNORMAL ATTENUATION PROFILE

As outlined in the first section of our paper, when our minds wander off there is an attenuation of a broad array of neurocognitive processing of stimulus events in the external environment. Similarly, some clinical populations have also shown attenuation of external processing akin to that observed during mind wandering states. Here, we consider the possibility that certain clinical conditions and mind wandering states may engage a common mechanism of sensory-motor attenuation, a possibility that aligns not just with observations of attenuation itself, but its association with activation of the brain’s default mode network (DMN; e.g., Gusnard and Raichle, 2001; Greicius et al., 2003). Specifically, not only has the DMN been shown to up-regulate activity during mind wandering (e.g., Mason et al., 2007; Christoff et al., 2009; Kirschner et al., 2012), but this up-regulation has been associated with down-regulation of activity in sensory cortices (e.g., Weissman et al., 2006). While abnormally heightened levels of DMN activity have been well-recognized in various clinical populations (e.g., Tian et al., 2006; Chai et al., 2011), here we stress their potential links to sensory-motor attenuation. While the associations we draw out remain admittedly speculative, by considering how patterns of sensory-motor attenuation may systematically vary with patterns of DMN activity, we may gain newfound leverage in our ability to understand both normal and pathological neurocognitive function.

On the one hand, ADHD is a psychiatric disorder characterized by inattention, hyperactivity and restlessness. Traditionally, one neuropsychological model of ADHD proposed that deficits of executive functions play an important role in the symptom manifestation of this disorder (e.g., Barkley, 1997; Willcutt et al., 2005). This model was supported by evidence of impaired performance on executive function tasks found in ADHD patients (e.g., Faraone and Biederman, 1998; Willcutt et al., 2005). Yet, not all ADHD patients exhibit executive function deficits, indicating that these deficits can explain but are not necessary for the symptoms to occur (e.g., Castellanos et al., 2006). If so, what else might account for the impairment of sustained attention in ADHD?

One hypothesis is that it may reflect functional abnormalities in the DMN; (Sonuga-Barke and Castellanos, 2007). For example, ADHD patients showed greater functional connectivity among regions within the DMN during rest as measured by fMRI (Tian et al., 2006), evidence suggesting a hyperactive default mode. Likewise, ADHD patients also showed greater variability in performance measures (Castellanos et al., 2005; Klein et al., 2006), which has been proposed to index attentional lapses in task performance (Sonuga-Barke and Castellanos, 2007). Collectively, these observations suggest comparatively higher levels of activity within DMN in patients diagnosed with ADHD.

Despite a hyperactive DMN however, ADHD patients showed a heightened propensity for distraction by external stimuli (e.g., Arnsten, 2006; Fassbender et al., 2009). From the perspective of sensory-motor attenuation, this suggests that there may be a reduction in the magnitude and/or extent of sensory-motor attenuation during DMN activation, relative to non-psychiatric populations. For example, whereas deviance detection stands out as the sole primary neurocognitive function identified to date that appears to be relatively preserved during mind wandering states (Kam et al., 2013a), the profile of “preserved” neurocognitive functions in individuals diagnosed with ADHD may be broader in extent and/or greater in magnitude than what has been normatively defined. Again, although speculative, this possibility represents a clinically-important hypothesis for future investigation.

Critically, however, abnormal profiles of sensory-motor attenuation – including both the range of neurocognitive processes involved and the magnitude of their attenuation – are not theoretically limited to *less* attenuation than what is normative. From a clinical standpoint, there could be pathologies tied to *increased *sensory-motor attenuation relative to non-clinical or mind wandering norms, a possibility that aligns with what has already been observed in some clinical populations.

In schizophrenia, for instance, hyperactivity and hyperconnectivity have both been observed in the DMN (Whitfield-Gabrieli et al., 2009; Ongür et al., 2010; Chai et al., 2011), akin to what has been found in ADHD patients. Yet at the same time, schizophrenia patients also show reduced top-down attentional modulation of external auditory signals, such that sensory-evoked N1 ERP responses are attenuated relative to non-psychiatric controls (e.g., O’Donnell et al., 1994; Salisbury et al., 2010). While to date these two findings have not been directly linked, they raise the possibility that this clinical population may show a broader array of sensory-motor attenuation effects, and/or a greater magnitude of these effects, relative to non-psychiatric norms. Regardless of whether that proves to be the case, the critical point is that the same general mechanism of sensory-motor attenuation engaged by mind wandering is also implicated in at least one class of neurocognitive disorders; further, these population-specific patterns of sensory-motor attenuation may be an important – and heretofore underappreciated – functional biomarker of the given neurocognitive pathology.

### ABNORMAL ATTENUATION PREVALENCE

Beyond mapping the profile of neurocognitive processes subject to sensory-motor attenuation during mind wandering and the magnitude of those effects, a second clinically relevant aspect of attenuation is in the prevalence of its engagement, or the proportion of waking hours spent attenuating the neurocognitive processing of the outside world. Normatively speaking, individuals report mind wandering almost half the time, regardless of whether its frequency is measured in the lab or a “real-world” setting (Smallwood and Schooler, 2006; Klinger, 2009; Killingsworth and Gilbert, 2010). Further, within the amount of time spent mind wandering, thoughts containing fantasy elements seem to occur about 25% of the time (Klinger and Cox, 1987). Here we consider systematic deviations in these normative prevalence rates as they relate to two different clinical populations, both of which concern identified abnormalities in decoupled thinking.

First, a pair of recent studies have proposed that a subset of the “normal” population engages in what can be described as excessive and/or compulsive fantasizing (Schupak and Rosenthal, 2009; Bigelsen and Schupak, 2011), or whimsical thoughts having content that is unlikely to occur or ever play out in the real world (Klinger and Cox, 1987). To these individuals, fantasizing occupies much of their personal life, and is seen comparable to an addiction. The content of their fantasies varies widely, but they fall under two main categories of those that are driven by fictional characters and those that involve the self in an aspirational context. Independent of the content, the fantasies are characterized by an intricate and elaborate level of detail (Schupak and Rosenthal, 2009; Bigelsen and Schupak, 2011). While they are fully aware that these fantasies are not real and are generally able to refrain from engaging in their fantasy world during work or school, they find the compulsion to indulge these fantasies uncontrollable when they are alone. The lack of control over their fantasy not only differentiates them from ‘healthy’ individuals, but also contributes to their distress over issues of time diverted from social activity and important relationships.

From a sensory-motor attenuation perspective, compulsive fantasy thus represents a deviation from the norm with respect to the prevalence rate of attenuation. Regardless of whether one is intentionally down-regulating the processing of external stimuli versus simply indulging one’s proclivity for fantasy, the byproduct is that compulsive fantasizers are engaging sensory-motor attenuation at rates that deviate from normative patterns. Moreover, to the extent that such decoupled thinking may in fact be addictive in this population (Schupak and Rosenthal, 2009; Bigelsen and Schupak, 2011), it raises the question of whether sensory-motor attenuation itself may be integral to the rewarding experience. That is, the addictive aspect of fantasizing may be as much about removing oneself from the outside world as it is about the actual fantasy playing out in one’s head.

A second clinical condition that can be seen as manifesting an abnormal prevalence rate for sensory-motor attenuation is major depressive disorder (MDD), a psychological disorder that is characterized by low mood, such as sadness, anxiety and helplessness, and a reduced level of physical activity, such as inability to sleep, or excessive eating. Of relevance, individuals experiencing depression have the tendency to ruminate in negative thoughts (e.g., Spasojevic and Alloy, 2001; Watkins and Teasdale, 2001), which in turn leads to negative moods. Given the vicious cycle of negative moods and negative thoughts (e.g., Segerstrom et al., 2000; Disner et al., 2011), the interaction between cognition and emotion appears to contribute to the symptoms of rumination in this disorder. Importantly, the tendency to perpetually engage in negative thoughts in depressed individuals suggests that they may show increased decoupling from the external environment, or an abnormal amount of time spent engaging sensory-motor attenuation.

Consistent with this possibility, depressed individuals showed increased activity and connectivity within the DMN (Greicius et al., 2007; Sheline et al., 2010). Moreover, several common symptoms of depression – lethargy and aversion to physical and social activity – also fall in line with this, as they reflect a decrease in engagement with the external environment. This population may also be characterized by impaired effortful task-related processing (Hartlage et al., 1993) – a problem that seems to be specifically induced by rumination (Watkins and Brown, 2002). These reports collectively suggest that depressed individuals are more likely to be in the default state, and once engaged, are more likely to stay there. This may be especially true for individuals with more severe symptoms and have become dysfunctional in their daily lives.

Taken together, compulsive fantasy and depression thus both appear to reflect an abnormal prevalence of the engagement of sensory-motor attenuation. While it is important to consider these neurocognitive pathologies in the context of attenuation profiles, there are clearly other factors involved specific to the disorder that lead some people to compulsively fantasize and others to uncontrollably ruminate. For example, emotion plays a crucial role in depression, while executive function capacities appear to contribute to ADHD and personality differences may modulate the severity of compulsive fantasies. The interaction between these factors and decoupling abnormalities may together account for more variability in the symptom manifestation and severity of each disorder. These findings highlight the value of considering subjective experiences in understanding the neural processes underlying clinical syndromes and neurocognitive pathologies.

## SUMMARY

In summary, our review emphasizes that decoupled thoughts are facilitated and maintained through an attenuation of a broad array of neurocognitive systems that are involved in responding to the sensory-motor environment. This attenuation serves the important purpose of buffering our internal trains of thoughts from external distractions and allowing one to fully engage in our decoupled thoughts, be they “normal” everyday content or be tied to a neurocognitive pathology such as major depression. We now turn to several points of consideration and future directions.

## POINTS OF CONSIDERATION

### SUBJECTIVE REPORTS OF ATTENTION

While subjective verbal reports provide a straightforward measure of one’s attentional state, a commonly raised concern with this particular method is that it may increase the risk of demand characteristics, and thereby potentially affecting the validity of the reports. There are several reasons why this concern may not be warranted. First, the proportion of on-task vs. mind wandering reports have been relatively consistent across studies regardless of the methodology used, whether participants provided a response verbally or through button press (Smallwood et al., 2008; Christoff et al., 2009; Kam et al., 2011; Kirschner et al., 2012). Second, several lines of research converge on systematic differences between these two attention states. These studies have revealed reliable differences in activation of neural regions (Mason et al., 2007; Christoff et al., 2009; Christoff, 2012), electrocortical processing of external stimuli (O’Connell et al., 2009; Barron et al., 2011; Kam et al., 2011, 2012), ocular patterns (Reichle et al., 2010; Schad et al., 2012), as well as behavioral performance (Smallwood et al., 2004, 2006; Kam et al., 2013a). As with any subjective measures in other areas of research, the possibility of demand characteristics remain; nevertheless, that demand characteristics alone may have contributed to the patterns of findings across mind wandering studies seem highly unlikely.

### RELATIONSHIP BETWEEN THE DEFAULT MODE NETWORK AND MIND WANDERING

The DMN is a resting state network of regions that includes the precuneus, posterior cingulate cortex, medial prefrontal cortex and bilateral temporoparietal junction, and has been shown to be more active at rest than during task performance (e.g., Gusnard and Raichle, 2001; Greicius et al., 2003). The functional significance of the DMN includes its role in self-referential thought (e.g., Northoff et al., 2006; Fingelkurts and Fingelkurts, 2011) and autobiographical memory retrieval (Svoboda et al., 2006; Kim, 2012). Given that mind wandering periods have been associated with activations of the DMN (Christoff et al., 2009; Kirschner et al., 2012), to what extent can research in DMN inform us about the functions of mind wandering? For instance, given the aforementioned roles of DMN and its relationship with mind wandering, one may speculate that we tend to engage in self-referential thoughts or autobiographical memory retrieval during mind wandering. Undoubtedly, more studies are necessary to determine the extent to which we can draw inferences about mind wandering based on research findings of DMN.

## FUTURE DIRECTIONS

### IS DECOUPLING AN INCIDENTAL OR NECESSARY PROCESS?

An important issue regarding the decoupling process concerns whether its occurrence is incidental or necessary. On the one hand, perceptual decoupling is thought to be necessary to support the continuity of internal thoughts (Smallwood, 2013). In contrast, Franklin et al. (2013) question the extent to which perceptual decoupling needs to be actively engaged to insulate these inner thoughts. While this debate is beyond the scope of this review, the literature we presented here appears to support the notion that an attenuation of external processing is necessary for the facilitation and maintenance of our internal trains of thoughts. This is in line with the notion that our executive resources are finite, which presumably limits the amount of stimuli we can attend to at any given point in time (Smallwood and Schooler, 2006).

### WHAT IS THE TIME COURSE OF MIND WANDERING AND ITS ASSOCIATED ATTENUATION?

An important and related question regarding the mechanism underlying this sensory motor attenuation concerns the temporal characteristic/order of internal thoughts and attenuation of external environment. That is, does the internal thought appear in consciousness first, and then the attenuation follows? Or does the attenuation occur first in order for the internal thought to creep into consciousness? This issue relates to a recent theoretical paper proposing a distinction between occurrence and process of self-generated thought (Smallwood, 2013). Specifically, the process that initiates an episode of self-generated thought is different from the process that maintains the integrity of that thought. Given Smallwood’s (2013) proposal that attenuation of external processing occurs to maintain the continuity of internal thoughts, one would predict the attenuation follows the initiation of the thought. However, this question remains to be empirically tested. To address this question would require the identification of the onset of internal thoughts and the temporal characteristic of the attenuation.

### CAN WE PREDICT AN INDIVIDUAL’S ATTENTIONAL STATE ONLINE?

Our research has shown that mind wandering is associated with changes in our neural responses to external events. Specifically, we found reductions in both sensory and cognitive level processing of external stimuli during periods of mind wandering (Smallwood et al., 2008; Kam et al., 2011), a disruptive effect of mind wandering that has been confirmed by other studies (O’Connell et al., 2009; Barron et al., 2011; Braboszcz and Delorme, 2011). Given these relatively stable neural signatures of mind wandering, can we reliably predict an individual’s attentional state based on their neural response in the moment?

One approach is to first establish a neural marker of mind wandering specific to the individual. That marker can then be used as an indicator of mind wandering when evaluating neural responses in real time on an individual-by-individual basis. That is, one could presumably compare whether the current incoming neural response matches the neural markers of mind wandering for that particular individual to determine whether their attention was focused externally or internally in real time.

Based on a similar approach, preliminary evidence has revealed that specific patterns of RTs during reading can be used to predict attention states online at the individual level (Franklin et al., 2013). Although these behavioral patterns are specific to the context of mindless reading, they nevertheless suggest the possibility of predicting one’s attention state in real time, without the need of individuals’ subjective reports. Therefore, future efforts need to be directed toward identifying neural markers to predict attentional states rather than simply characterizing neural responses associated with subjectively reported attentional states.

### IS THERE A NEURAL NETWORK THAT REGULATES THESE ATTENTIONAL FLUCTUATIONS?

Both theoretical models (Sonuga-Barke and Castellanos, 2007) and empirical evidence (Smallwood et al., 2004, 2008; Christoff et al., 2009; Kam et al., 2011) point to the existence of slow fluctuations of attention between inner thoughts and external environment. There are two main stages involved in these fluctuations, the processing of which seem to require the effort of a superordinate system. First, given that internal and external inputs are always competing for executive resources (Smallwood and Schooler, 2006), the importance of each stream is evaluated and compared. Second, the stream deemed as more important presumably enters consciousness. As distinct as these experiences are, the transition between these attention states at times appear to be seamless. So is there a network of regions that regulates this evaluative process and facilitates the transitions?

One potential candidate is the salience network, comprised of the anterior insula and anterior cingulate cortex, which was proposed to distinguish the most salient stimuli among internal and external inputs in order to guide behavior (Menon and Uddin, 2010). Consistent with the above model, the frontal parietal network (FPN), which consists of the rostrolateral and dorsolateral prefrontal cortex, anterior insula, dorsal anterior cingulate cortex, precuneus, and the anterior inferior parietal lobule, has also been proposed to serve a similar function (Smallwood et al., 2012). This network of regions was suggested to act as a “global workspace” (Baars, 1988), which adjudicates between internal stimuli and external stimuli competing for access to consciousness (Smallwood et al., 2012). Of relevance, the FPN was hypothesized to work with the DMN in facilitating internally directed thoughts by protecting them from disruptions by the external environment. Evidence supporting this hypothesis comes from neuroimaging research showing that parts of the FPN were activated during periods of mind wandering (Christoff et al., 2009). Taken together, emerging evidence suggests that these networks are potential candidates as regulatory bodies of attentional fluctuations; nevertheless, the exact function of these networks as well as the direction of causality between these networks and the DMN is yet to be determined.

### WHY DO WE MIND WANDER?

Much of our discussion of mind wandering concerns its disruptive effects on processing of external stimuli resulting from decoupling our executive resources from the environment. This disengagement from the external world plays an important role: it allows us to focus in our inner world. But what exactly is the purpose of engaging in our own trains of thoughts?

Several lines of research have shed light on the functionalities of mind wandering. For example, both task-unrelated thoughts and the DMN have been positively associated with levels of creativity (Baird et al., 2012; Ellamil et al., 2012). In particular, the greatest improvement in creativity test performance was observed in the experimental condition that elicited highest levels of mind wandering (Baird et al., 2012). This finding suggests that periods of mind wandering may act as incubation intervals necessary for creative solutions to be generated, a positive feature that highlights the potential value of mind wandering.

Moreover, the thoughts that individuals engage in tend to be oriented toward the future (Smallwood et al., 2011). Consistent with this finding, the neural regions implicated in future thinking (Schacter et al., 2007) overlap with regions associated with mind wandering (Mason et al., 2007; Christoff et al., 2009). Interestingly, a significant portion of the content of our inner trains of thoughts has been associated with our current concerns (Klinger and Cox, 1987). This pair of findings suggests that thoughts about current concerns during mind wandering may be used to plan future behavior.

In addition, mind wandering may restore attentional capacity by offering an escape from the task at hand. Based on the Attention Restorative Theory, effortful attention can become exhausted over time in an urban environment, but can be restored in an environment that facilitates “fascination” or effortless attention (Kaplan and Kaplan, 1989). For example, a natural environment has been rated as highly effective in restoring attention (Herzog et al., 1997), an effect that was confirmed by findings that a walk in nature allows for recovery of directed attentional abilities (Berman et al., 2008). While a walk in nature may not always be plausible, might a “mental walk” away from the current task provide a similar albeit potentially smaller effect of attentional restoration?

Accordingly, it appears that mind wandering is not only associated with costs but also with benefits, (e.g., Smallwood and Andrews-Hanna, 2013), an issue that seems to depend on the context in which it occurs, as determined by a host of variables that transcend the actual attenuation itself. While these adaptive features of mind wandering shed light on its functionalities, they do not directly address the question of the purpose of mind wandering. Both theoretical models, perhaps from an evolutionary standpoint, and future research are necessary to elucidate the purpose of this ubiquitous experience.

### IS IT POSSIBLE TO CONTROL THESE FLUCTUATIONS OF ATTENTION? IF SO, HOW?

Despite the negative connotations commonly associated with mind wandering, its aforementioned functionalities make it apparent that the issue is not in the experience itself, but instead lies in the control over the occurrence and duration of this experience. That is, if we engage in our own thoughts in a safe environment when disrupting the task at hand is not detrimental, then mind wandering can afford multiple adaptive functions. Accordingly, it appears that being in control of when and for how long to mind wander is the key to capitalizing on its benefits while avoiding its disruptive effects. Several approaches that have recently emerged with the aim to enhance the control over one’s attention include mindfulness training and biofeedback. Preliminary evidence suggests that mindfulness training does indeed improve one’s sustained attention (Jha et al., 2007; Tang et al., 2007). Notably, additional research is needed to determine whether these techniques only prolong attention once engaged, or whether they actually allow us to exert explicit control over what enters consciousness.

## CONCLUSION

Taken together, decoupled thoughts are associated with an attenuation of neurocognitive systems involved in processing stimuli in the external sensory-motor environment in order to adaptively guide our behavior. That these decoupled thoughts occupy much of our awake time, and are tied to a variety of neurocognitive functions highlight the importance of research in mind wandering. Future studies are necessary to understand better these temporal fluctuations of attention, as they are vital to both normal and pathological neurocognitive functioning.

## Conflict of Interest Statement

The authors declare that the research was conducted in the absence of any commercial or financial relationships that could be construed as a potential conflict of interest.
